# Illness Appraisals and Depression in the First Year after HIV Diagnosis

**DOI:** 10.1371/journal.pone.0078904

**Published:** 2013-10-25

**Authors:** Judith Tedlie Moskowitz, Judith Wrubel, Jen R. Hult, Stephanie Maurer, Michael Acree

**Affiliations:** Osher Center for Integrative Medicine, University of California San Francisco, San Francisco, California, United States of America; The University of New South Wales, Australia

## Abstract

Illness appraisals provide important context to help understand the way individuals cope with chronic illness. In the present study, a qualitative approach to the analysis of HIV diagnosis experience narratives in a sample of 100 people newly diagnosed with HIV revealed five groups that differed in their initial illness appraisals: *HIV as Chronic Illness*, *Concern about Dying, Stigmatization*, *Threat to Identity*, and *Other Threats Overshadow HIV*. When compared on quantitatively measured depressive mood, the groups differed on level and trajectory over the course of the first year post-diagnosis. Although the experience of living with HIV has changed significantly with the advent of effective Antiretroviral Therapies (ART), there were a number of similarities between the appraisals of this group of participants who were diagnosed post ART and groups who were diagnosed before ART became widely available. Posttest counselors and other HIV service providers should take individual differences in illness appraisals into account in order to help newly HIV-positive clients manage their healthcare and cope adaptively with their diagnosis.

## Introduction

Each year millions of Americans receive a diagnosis of a serious illness such as cancer, heart disease, rheumatoid arthritis, hypertension, or HIV (http://www.cdc.gov/nccdphp/). Following quickly on the heels of a diagnosis are a host of potential illness and treatment-related stressors, including necessary changes in health behaviors, having to make decisions with incomplete information, unpleasant side effects of treatment, and coming to terms with a new identity as someone with a serious illness (e.g., [1,2].) In the face of these demands, people living with serious illness can experience elevated levels of depression, anxiety, and even symptoms of posttraumatic stress disorder [3–5]. 

Much of the research on adjustment to serious illness has focused on *coping* as the primary determinant of psychological well-being. Although a number of studies have demonstrated an important role for coping in adaptation to diagnosis with a serious illness, a great deal of variance in psychological response remains unexplained by coping responses [6–9]. According to Stress and Coping Theory [10], *appraisal*, the individual’s perception or interpretation of the significance of an event for his or her well-being, is a critical component of the stress and coping process. An appraisal of an event as personally significant and as taxing or exceeding the resources of the individual is referred to as a “stress appraisal.” Stress appraisals are associated with emotions: negative emotion in response to threat or harm, a mix of positive and negative in response to challenge. Stress appraisals prompt coping directed at managing the problem and regulating emotion. If the event is resolved favorably, a positive emotional state is the result. If the event is resolved unfavorably or if it is unresolved, a negative emotional state results and the coping process continues through reappraisal and another round of coping. The appraisal/coping process is transactional in that it involves a dynamic relationship between the person and the environment in a given context. 

 Illness appraisal encompasses the meaning that the illness and its sequelae have for the individual’s future health and well-being and may include beliefs about the course and consequences of an illness, and the experience and interpretation of symptoms [11,12] . Illness appraisals are central to understanding how individuals respond emotionally, cope with, and adjust to chronic illness. The present study focuses on illness appraisals at the time of HIV diagnosis.

### Illness Appraisals

Health researchers have approached illness appraisals in various ways including as illness meaning [11,13–16], illness representations (e.g., [17]), personal models of illness [18], common-sense models of illness [19,20], illness perceptions [21], and implicit models of illness [22]. Illness appraisals have been assessed both quantitatively (e.g., Meaning of Illness Questionnaire[13] IPQ [23]), and qualitatively [24–26]. This previous work demonstrates that illness appraisals change over time [20], influence decision-making and subsequent health behaviors [27,28], may be central to the understanding of how the individual copes with chronic illness [14,16,26,29], and can be modified through targeted interventions [30,31]. 

### Qualitative approaches to understanding HIV illness appraisals

Since the first cases of AIDS were identified in the early1980s, the experience of living with HIV has changed from a largely acute illness that for many was imminently terminal, to a chronic illness that can be managed with more effective antiretroviral (ART) medications that came into widespread use in the mid 1990s [32]. Early in the epidemic, researchers interviewed people living with HIV with the aim of describing their illness appraisals. For example, based on clinical interviews with 19 HIV- positive gay men, Schwartzberg [33] developed a typology of personal meanings or appraisals of HIV. The four appraisals were transformation (HIV had influenced their life and changed them in profound ways), camouflage (appearing much like the transformation group but not seeming to have been truly transformed), rupture (HIV shattered their life meaning and they had not yet been able to rebuild), and impassivity (indifferent to the impact of HIV on their lives).

In another qualitative study of HIV illness appraisals conducted before the ART era, 57 men living with HIV were interviewed monthly over a period of years and asked to report recent HIV-related stressful events as well as recent positive events. Based on these interviews, Moskowitz and Wrubel [24] described five types of HIV illness appraisals: “Future Focus,” in which participants appraised HIV-related stressful events in terms of implications for their death, despite the fact that they were relatively healthy; “Detached,” in which participants appeared to experience HIV-related stressful events at arm’s length, similar to Schwartzberg’s [33], Impassivity group; “Stigma” in which participants felt stigmatized by their HIV but also held stigmatizing attitudes toward others living with HIV; “Outward Focus,” those who attended to others and offered as well as received social support; and “Aware/Avoid” in which participants valued the cognitive awareness of HIV and its implications, but at the same time spent a lot of effort trying to avoid the negative emotions that accompanied such awareness. 

This shift in the nature of HIV from an imminently terminal to a more manageable chronic illness following the widespread use of ART could be expected to change how people who are living with HIV appraise the illness, but a significant shift in appraisals across the board is not necessarily evident in the research literature. For example, the transformative or wake-up call appraisal appeared both before and after the widespread use of ART [26,34–37]. The view of HIV as a death sentence found in the Schwartzberg [33] and Moskowitz and Wrubel[24] data was still evident for some participants post-ART[34,35,38,39] although this was not a dominant appraisal for everyone (e.g., [40]). Flowers and colleagues[41] interviewed HIV-positive gay men diagnosed after the introduction of ART regarding their illness appraisals in the 4 years after HIV diagnosis. The in-depth interviews captured within-individual changes in appraisals after diagnosis and reflected both similarities and differences compared to the predominant pre-ART appraisals. For example, whereas there was still some evidence of perceptions of HIV diagnosis as a death sentence, this appraisal tended to become less dominant over time. Diagnosis was still associated with significant levels of distress, but the source of the distress appeared to have shifted from a primary concern with imminent death to more social and psychological factors such as disclosure and implications for identity. 

Another factor that could be expected to influence illness appraisals is the recency of diagnosis with HIV. Valle and Levy [34] interviewed 80 African American injection drug users who had tested HIV-positive in the past 3 to 18 months. Participants were asked to report their initial reactions to the HIV test results and “what they perceived being diagnosed with HIV meant.” Based on their narrative responses, the authors found three mutually exclusive interpretations of the HIV diagnosis. Forty-five percent of the respondents interpreted testing positive as “just another problem,” another difficulty in a series of negative life events. Thirty-five percent felt testing positive was a “wake up call” that changed their life for the better, and 15% interpreted HIV as a death sentence, which led to increased self-destructive and pleasure seeking behaviors. The participants in this study were not followed longitudinally, however, so it was not possible to determine whether these early HIV illness appraisals were prospectively associated with psychological well-being. 

The aim of the present study is to describe illness appraisals in 100 people who had been diagnosed with HIV within the past 2 months and determine associations of initial appraisal with depressive mood over the course of the first year after diagnosis. Data were collected well after the advent of highly active antiretroviral therapies that significantly extend the lives of people with HIV. Using a stress and coping theoretical framework [10,42] we analyzed participant narratives of testing positive for HIV, grouped participants based on the appraisals expressed in these narratives, then examined whether these initial HIV appraisals were associated with depressive mood over the subsequent 12 months. 

## Methods

All procedures were approved by the Committees on Human Research at institutions where recruitment or interviewing of participants took place: UCSF, Alameda County Medical Center, and Children’s Hospital and Research Center in Oakland, CA. Signed informed consent was obtained from all participants. 

### Participants

Participants were 100 HIV+ men and women who were enrolled in the CHAI (Coping, HIV, and Affect Interview) Study, a longitudinal cohort study of people newly diagnosed with HIV. A convenience sample of participants was recruited in the San Francisco Bay area between 2003 and 2008 through local HIV testing sites and clinics. This subset of the full CHAI study consisted of the first 100 enrolled and was selected based on data and resource availability at the time the analyses were conducted. Given that 100 is a relatively large sample for qualitative analysis, we felt it was justified to go forward and conduct the analyses on this subsample. To be included in the study participants had to (a) have been informed they were HIV positive within the previous 8 weeks, (b) speak English, (c) be 18 years or older, (d) have the ability to provide informed consent to be a research participant. HIV status and date of disclosure were verified with the clinic from which they were recruited. If participants were not recruited directly from a clinic site, their HIV status and disclosure date were verified through the clinic where they were tested or through their physician. Evidence of severe cognitive impairment or active psychosis resulted in exclusion from the study. Each participant was given $30 at the completion of each interview. This amount was comparable to incentives provided by other studies in the San Francisco Bay area at the time and was considered appropriate by the Committees on Human Research who reviewed the study for human subjects approval. 

### Procedures

Data for the present analyses come from a larger longitudinal study in which participants completed 2 hour interviews seven times over the course of 18 months (at approximately 1, 2, 3, 6, 9, 12, and 18 months after diagnosis)[43–46]. In the present study we report qualitative data on illness appraisals collected as part of the baseline interview (month 1), and quantitative data on depressive mood from interviews conducted at baseline, and months 2, 3, 6, 9, and 12. 

Baseline interviews were conducted by masters-level trained interviewers with participants who were within 8 weeks of receiving an HIV diagnosis. Interviews were conducted at hospital clinics or at clinical research centers. Participants were asked to tell their story of testing positive for HIV: how they came to be tested, how they got their results, who told them, how they were told, how they felt when they heard about it, and how they've been doing since then. The interviewer used the following probes, if necessary, in order to elicit the story: “What brought you to be tested?” “When did this happen?” “How did you get your results?” “What were you feeling?” “What made you feel that way?” “Was testing positive stressful for you?” “What did you want to do when this happened?” “What did you actually do?” and “Why did you choose this particular action?” Interviews were audiotaped, and qualitative portions were transcribed for analysis. 

We also include quantitative data on depressive mood collected in 6 assessments conducted over the course of the first year after diagnosis. This study focuses on data from the first 100 participants who completed the baseline interview.

### Narrative Analysis

We used an interpretive phenomenological approach [47] to analysis of the narratives. We approached the analysis of the narratives first by coding each individual interview, and then by comparing the interviews. The aim of coding cases was to identify aspects of the coping transaction as they appeared for each participant in order to be able to examine each aspect across all interviews. As a first step, four authors (J.T.M., J.W., J.H., and S.M.) used a team-based approach to developing codes and coding the narratives [48]. The coding protocol was developed based on previous illness appraisal work [24] and review of the interviews from 20 participants. We then used the protocol to code the interviews of the other 80 participants. 

 The Stress and Coping theoretical framework [10], provided an initial starting point for coding and focused on three overarching categories: appraisal, coping, and emotion. The particular codes were developed as they arose from each narrative. The stressful event was the same for all participants, namely the experience of testing positive. For each stressful event narrative we coded the appraisals of what specifically made receiving their diagnosis stressful for the participant, their emotions, and coping. Twenty interviews were sufficient to saturate the codes. The 20 cases were reviewed for consistency of coding. The remaining 80 cases were then divided among the four coders, coded, and exchanged for verification. Disagreements were resolved through discussion. We used Atlas.ti, a qualitative data management software, to manage the data and track coding and analyses. 

#### Coding Scheme

Our final coding scheme consisted of 154 codes grouped into 11 parent, or overarching, categories. For the purposes of the present analysis, we focus on the three of these parent categories: Appraisal, Coping, and Emotion.

#### Appraisal

Each event was coded for the appraisal of what was harmed, threatened, or challenged by testing positive. The question for the coders was specifically, “What was at stake for the participant in the narrated encounter?” In other words, what, according to the participant, made the event stressful? There were a total of 32 appraisal codes, and each event could be coded with more than one appraisal. See Table 1 for a list of the most common appraisals and their total frequency. 

**Table 1:** Most frequent stressful event challenge/threat codes.

**Code**	**What is appraised as a challenge/threat?**	**Example**	**F Frequency**
SELF VIEW	How they see themselves in regards to getting HIV or being someone with HIV.	“I just couldn’t believe it… ’cause I never thought that I’d be HIV-positive.”	42
OWN DEATH	Participant's concern about his/her own death.	“…but the time is going to come when I’m gonna die anyway.”	34
HEALTH CONCERN	Participant's concern for his/her own health.	“I’m still deciding whether to take a certain medication…there’s some stress around it…because it’s toxic...”	32 32
STIGMA	Concern over discrimination or feelings of stigmatization.	“a couple people know, but I don’t want to tell my friends because I feel really shameful that… that here I am HIV positive… and the stigma of that…”	31
DISCLOSURE	Concerns over disclosing, including not wanting to worry others.	“…there’s people in our lives we care about…I had to tell them about it…and it wasn’t so much for me, but I had to like, to like, watch them react.”	29
FUTURE HEALTH	Concern over own future health. Can be positive or negative outlook but wanting to stay healthy.	“Now he brought me this shit and I don’t like it. Now I’ve got to deal with it for the rest of my life.”	20
SOCIAL SUPPORT	Concern about social support, including support by partner and potential loss of support.	“…it’s just strange since I can’t get any support from him right now…so that’s frustrating.”	19
FUTURE PLAN	Concern about possible future plans.	“you know, what about these plans I had for my life, things I wanted to do…was it all for nothing?”	17
NOT INFECT OTHER	Not wanting to infect other people with HIV	“I couldn’t think about anything else but that because I was afraid that my partner was infected…”	12
APPEARANCE	Concern about personal attractiveness	“I don't want to look - not that I think I'm a supermodel now, but you know, definitely not that, you know, right now”	11
SOBRIETY	Desire to stay sober/drug free	“…because nothing’s more important to me than staying sober and keeping that creeping crystal meth outta my life…”	11

Note: Frequency is number of participants (of 100) who noted the stake in their diagnosis narrative. A participant could have multiple challenge/threat appraisals coded in their narrative.

#### Coping

We identified 8 forms of coping in the narrative data that matched those found in the Ways of Coping Checklist [49], a widely used quantitative measure of coping (accepting responsibility, behavioral escape/avoidance, cognitive escape/avoidance, distancing, confrontive coping, seeking social support, positive reappraisal, and planful problem solving). We found additional types of coping reported in the narratives including blaming another person, creating other positive experiences, focusing on something positive, humor, offering social support, praying, reassuring oneself, self-restraint, and venting. A narrative could be coded for as many types of coping as were reported, and often a single stressful event was associated with multiple types of coping. 

#### Emotions

Emotions mentioned in response to the “What did you feel?” probe as well as other emotions mentioned as experienced in the context of the event were coded verbatim, then grouped into positive, negative, or other (i.e. surprise). We defined positive emotions as those with a generally pleasant valance such as grateful, love, relieved, and hopeful and those with a generally negative valence such as afraid, disappointed, guilt, and shame. 

### Thematic Illness Appraisal Comparison

We grouped participants based on common themes that shaped what was understood as stressful about testing positive (appraisal), what showed up as possibilities for coping, and their emotional responses at the time of the encounter. The resulting typology reflected the different ways in which the participants appraised their HIV diagnosis. The identification of and assignment to a group was conducted jointly by the qualitative team (J.T.M., J.W., J.H., & S.M) and disagreements were resolved through discussion. Each participant was assigned to only one group. 

*Depressive* mood was measured at each assessment point with the CES-D [50] a 20-item self-report scale that assesses aspects of depressive mood that occurred during the previous week. Responses are on a scale ranging from 0 (rarely or none of the time) to 3 (most or all of the time). 

### Quantitative Analysis

In order to compare the qualitatively derived appraisal groups on depressive mood over the course of the first year after diagnosis, we conducted a repeated measures analysis of variance using SAS PROC MIXED. The mixed model allowed us to include participants with incomplete data in determining whether the groups differed on average level of depressive mood and change in depressive mood over time. 

## Results

On average, the participants were 39.4 years old (range from 19 to 57). Seventeen percent reported high school as their highest level of education. Twenty-eight percent completed some college, 30% had a college degree, and 24% had some education beyond college. The median household income was between $20,000 and $30,000 per year. Fifty-five percent of the sample was White, 26.6% African American, 8.5% Latino, 5% Asian or Pacific Islander, and the rest reported mixed race/ethnicity. The majority of the participants were male (92%) and identified as gay/bisexual (86%). Mean CD4 count was 467.6 (median 424, range 9-1300). 

Based on our qualitative analysis, participants were placed into five mutually exclusive groups that differed on their thematic illness appraisal at the time of testing positive for HIV: *HIV as a Chronic Illness* (*N* = 24); *Concern about Dying* (*N* = 23); *Stigmatization* (*N* = 20); *Threat to Identity* (*N* = 19); and, *Other Threats Overshadow HIV Diagnosis* (*N* = 14). These descriptions are based solely on the narrative data. 

### HIV as Chronic Illness

“It was like, ‘Okay, it’s just HIV. It doesn’t mean I'm going to die.’”

This group (*N* = 24) appraised their diagnosis as a serious danger to their health, and that appraisal dominated their narrative. Although they appraised HIV as a threat, they also viewed it as a chronic illness that could be managed.

*It’s devastating, to say the least, when somebody tells you something like that, but, I guess there’s part of me that says, well, they have medications that I can take that will add years onto my life. If I take care of myself I’ll be all right. And so, that’s why I'm not terribly down in the dumps right now because I believe if I take the medicines and take care of myself that I’ll be okay.*

These participants incorporated a proactive approach toward coping with HIV. This approach included seeking information generally about HIV and HIV treatments, as well as information specific to their own disease (e.g., CD4 counts), making follow-up doctor appointments, finding out about studies they could enroll in and programs for helping people with HIV, and talking to others living with HIV about their experiences.

*Interviewer: And what did you feel like doing?*

*Participant: Treating it just as soon as possible. Getting some sort of treatment because I knew that HIV could be treated, and it’s like, “Okay, I've got to move on and get this treated so that I can feel better.”*

Because the appraisal of HIV as a chronic illness evoked problem-focused coping directed towards getting information and seeking medical care, the participants in this group had the possibility of experiencing positive affect in response to the effectiveness of their coping. In the following narrative, the participant describes some of the positive emotions he experienced in the course of dealing with his diagnosis:

*Part of it has been stressful because I have had to really think about health and that I have to really be careful about things even more so now, but in other ways it has been a* ***relief*** *to know that I have resources that have opened up to me that weren’t available before. I'm really* ***grateful*** *because a lot of the resources that were open to me---the people that I've met already have just been really supportive and really sincere and genuine.*

### Concern about Dying

“When I tested positive it was very clear to me that I would die.”

This group (*N* = 23) initially appraised their HIV diagnosis as meaning they would die soon. This appraisal by 19% of our sample belies the current notion that with the advent of ART people no longer view HIV seropositivity as a “death sentence” (e.g., Heckman, 2003; Reiter, 2000).

*There’s no turning to the left or to the right. I see the end and the end means … well, basically it’s my death.*

Strong negative emotions of sadness, fear, upset, and anger accompanied the participants’ appraisal that HIV meant they would die. Reappraisal was the most typical coping used to manage this threat. If the participants were able to hear and believe reassurances that HIV was now a manageable chronic illness, they modified their initial dire appraisal.

*[The doctor] is like you’re going to live a very long, pretty normal, healthy life. You’ll probably live to be about 70. He’s like, so I don’t want you to be worried about that. And when I heard that I was like, oh, 'cause I didn't know, you know, people were living that long with this.*

Participants in this group used reappraisal as a way to cope with their thematic illness appraisal more consistently than any other group (79%). Reappraisal is a way of indirectly managing the stress appraisal by lessening the sense of threat and thus reducing the negative emotions. This was sometimes adaptive if it had the effect of refocusing the person on engaging in direct action (like seeking information, making doctor appointments) or in gathering social support. Within the first interview, many participants shifted back and forth between their initial appraisal to reappraisal and to their initial appraisal again. 

### Stigmatization

*“I was guilty and ashamed 'cause there’s a stigma about it.”*

Concerns about stigma dominated this group’s (*N* = 20) stress appraisals. Stigma for HIV seropositivity has multiple meanings. In this group, these meanings included stigma concern based on experience, fear of being stigmatized by others, and internalized stigma. Participants in this group all had a generalized feeling that they would be stigmatized by others for being HIV positive.

*I was guilty and ashamed 'cause there’s a stigma about it. Although one in three gay men in San Francisco are positive, there still is a stigma with that, and it just put me in that group.*

Some participants had already had direct experience with HIV stigma, which added to their fear of being stigmatized by others.

*My parents don’t know yet. My sister has it, has HIV. And my dad was saying, “Oh, your sister got this, and I don’t want her to talk to me no more.”*

In addition, some participants had internalized stigma that brought with it strong negative emotions and negative feelings about the self.

*Interviewer: So how did you feel when you got your results?*

*Participant: Like a diseased whore. I felt like a pariah.*

One direct consequence of an illness appraisal of stigma was the participants’ reluctance to disclose their change in serostatus and the resulting lack of social support for the very stressful experience of testing positive for HIV.

*You can hear it on the bus, “Ooh, look that faggot, he pro’ly got AIDS.” So I haven’t told anyone, I’m sharin' this only to myself.*

The need to keep their diagnosis secret meant that participants in this group could not seek out the same informal social support as participants with other illness appraisals. It also affected their sexual risk behavior because of a reluctance to disclose to sex partners. 

 The *Stigmatization* group expressed more negative emotion in their narrative accounts than the other groups. An indication of the intensity of the negative emotion felt by this group is that half of the 10 participants who said they’d considered suicide when they received their HIV diagnosis are in this group.

### Threat to Identity

“It was like seeing things in a very new way. You know, my identity was being negative.”

These participants (*N* = 19) did not see themselves as the type of person who would ever get HIV and receiving an HIV diagnosis was a threat to their self view. Some participants simply didn’t believe they were in a risk category.

*I couldn’t believe that it was me that got those results? I remember thinking over and over again, “How did I test positive? Everyone else tests positive.” I had always been safe. So that kept goin' through my head--I couldn’t believe it.*

Other participants had developed a sense of invulnerability because of long histories of testing negative despite risky behavior

*I had come to a place where I think I had taken so many risks at times with positive partners that I just thought it [would*]* never happen to me. I feel like I’d possibly been exposed to HIV many times since 1990. Sort of disbelief because I’d felt, like, invincible.*

### Other Threats Overshadow HIV Diagnosis

“I have other things that are way more stressful than AIDS.”

Although, generally speaking, a diagnosis of HIV serostatus constitutes a stressful event, for these participants (*N* = 14), the event was not appraised as the most stressful event currently unfolding in their lives.

*AIDS can be controlled but my wife can’t. Everything was all piled up on me at that particular moment. The syphilis, not knowin' if there was any damage, 'cause I couldn’t see out of the right eye. So AIDS didn’t really bother me 'cause I know that you can take care of it nowadays.*

The circumstances in these participants’ lives were all extreme, like being hospitalized with other serious illnesses unrelated to HIV. One participant had uncontrolled epileptic seizures; another was hospitalized with a stroke. Other participants were dealing with trying to find food and shelter.

*This whole thing [i.e., testing positive for HIV*]* has been kind overshadowed by something else. You see these scars? This man that I’d never known in my life broke into my room while I was asleep and tried to kill me!*

Because of the need to deal with these more pressing threats, these participants did not engage in the proactive coping of getting information about HIV in general, or their level of disease in particular, or set up regular doctor visits, or seek practical and/or emotional support for their diagnosis. 

#### Quantitative Analyses

The five appraisal groups did not differ on race/ethnicity, education, gender, or sexual orientation (See Table 2). There was a significant difference on age. Participants in the *Other Threats Overshadow Diagnosis* group were significantly older than those in the *Stigmatization* and *Threat to Identity* groups (*F* (4,95) = 2.52, *p* = .046). Furthermore, the *Stigmatization* group had significantly higher levels of CD4 at study entry, compared to the other four groups (*F*(4,92) = 2.56, *p* = .04). 

**Table 2:** Descriptives by group.

	HIV as chronic illness (N=24)	Concern about dying (N=23)	Stigmatization (N=20)	Threat to Identity (N=19)	Other Threats overshadow diagnosis (N = 14)
Race/Ethnicity					
White	50	42.9	63.2	28.8	69.2
African American	25	38.1	31.6	11.8	23.8
Latino	16.7	9.5	5.2	23.5	0
Other	8.3	9.5	0	5.9	7.7
Age	41.9^b^	39.1^b^	35.5^b^	37.8^b^	43.4^a^
Education (years)	12	11.9	11.6	11.6	11.7
Sexual Orientation (% identifying as gay or bisexual)	96%	74%	90%	89%	78%
CD4 at entry into study	424^b^	445^b^	642^a^	440^b^	376^b^

Note: cells with different superscripts differ significantly from each other

**Figure 1:**
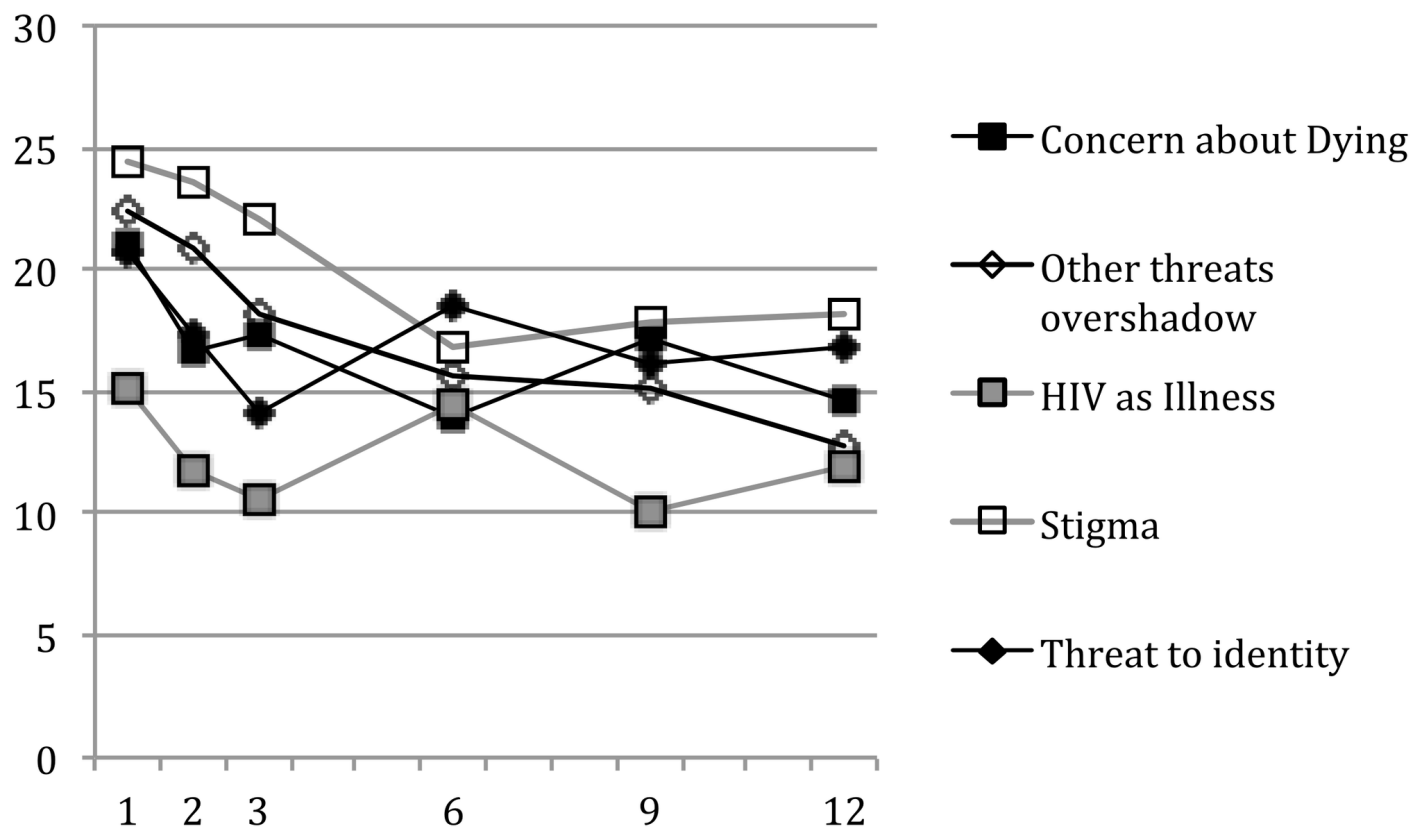
CES-D Score at each interview by HIV illness appraisal group.

The mean CES-D scores by group over the 12 months after diagnosis are in Figure 1. Of note, a score of 16 or above on the CES-D is considered at risk for clinical depression[50]. With the exception of the *HIV as Chronic Illness group*, all the groups had mean scores above this cutoff at the initial assessment. *Stigmatization*, *Other Threats Overshadow Diagnosis*, and *Concern About Dying* groups remained near or above this cutoff, even a year after diagnosis. There was a significant difference among the groups in CES-D level (*F* (4,95) = 3.38, *p* = .012) and a marginally significant difference in trajectory (appraisal group by months interaction *F* (4,386) = 2.24, *p* = .06). The *Stigmatization* group had a significantly higher level of CES-D overall compared to the *HIV as Chronic Illness* group and the *Other Threats Overshadow Diagnosis* group declined significantly more (slope = -.92 points/month) than the *HIV as Chronic Illness* group (-.20) or the *Threat to Identity* group (-.10).

## Discussion

This is one of the first large scale qualitative studies to examine illness appraisals in people who were newly diagnosed with HIV. Capturing these early appraisals may be uniquely helpful for HIV services providers, particularly those working with patients who are receiving an initial HIV diagnosis, as well as researchers interested in the process of adjustment to a serious illness such as HIV. HIV treatment guidelines increasingly recommend treatment with ART soon after diagnosis and close monitoring of viral load and other indicators of disease progression [51]. As the treatment landscape, and corresponding attitudes from providers and patients evolve, an understanding of the patient’s HIV appraisal can provide insight into the barriers to optimal engagement in care and adherence to ART and, potentially, help to reduce these barriers[26]. 

According to Stress and Coping Theory [10], appraisal of an event determines the emotional response. When receiving a diagnosis of a serious illness, the individual’s appraisal of the personal significance of the illness similarly influences subsequent psychological adjustment. Our qualitative analysis of participant narratives regarding their HIV diagnosis experience revealed five distinct ways in which a new HIV diagnosis is appraised in the era of effective medications that have significantly extended the lives and improved the health of people living with HIV. 

Despite advances in HIV treatment, 23% of our sample initially interpreted their diagnosis as foreshadowing a significantly shortened life, meaning that they would die from HIV. This appraisal of HIV was frequent prior to the advent of antiretroviral therapies (e.g., “Future Focus” in Moskowitz & Wrubel [24]), but a number of studies show that some continue to hold this appraisal despite widespread availability of these more effective therapies (e.g.,[34,35,38,41]}). The mean level of depression in this group we labeled *Concern about Dying* was high at baseline, and, although it decreased somewhat over time, as a group their mean score remained elevated, nearing the cutoff considered at risk for clinical depression. 

Similar to the participants in the study by Valle and Levy [34], who appraised HIV as “just another problem,” our *Other Threats Overshadow HIV* group had other serious life events that they were coping with: HIV was not their primary concern. Interestingly, they also tended to have generally lower levels of depressive mood compared to all except the *HIV as Chronic Illness* group. People who appraise an HIV diagnosis as less stressful than other things in their lives may be particularly difficult to engage in care because their focus is not on their HIV. Interventions that include disease-specific education may be beneficial (e.g.,[31]) but increased awareness of the challenges of HIV may also increase levels of distress, however, so should be implemented alongside information on how to cope with these challenges in order to avoid alienating the patient from engaging in appropriate healthcare. 

In the early years of the epidemic, HIV was a highly stigmatized disease and HIV illness appraisals reflected this high level of stigma [24]. Twenty percent of our current sample had *Stigmatization* as their main appraisal at the time of diagnosis. This appraisal reveals the persistence of stigmatizing attitudes toward others living with HIV as well as internalized stigma and fear of others’ negative responses to their diagnosis[38,41,52]. In this group, depressive mood was high at baseline and dropped some over time, but remained higher than all the other appraisal groups. These deeply held feelings of stigma may prevent people who hold this appraisal from seeking support from others and reduce the likelihood that they will engage in optimal levels of care. On the other hand, the *Stigmatization* group had significantly higher CD4 at baseline compared to the other four groups, indicating, perhaps, that they were tested earlier in the disease course. This hypothesis contradicts the possibility that people who hold stigmatizing attitudes would be less likely to engage in appropriate health care. We do not have data in the present study to address this speculation, however. Future work should consider the complex role that stigma may play in encouraging or discouraging optimal levels of care. A number of studies have begun to consider the important issue of stigma, both in HIV [53,54] and across other diseases as well [55,56]. 

The group that held the *Threat to Identity* appraisal was similar to the *Stigmatization* group in many ways. Both groups see the HIV diagnosis as a negative reflection of the kind of person they are. In the case of the threat to identity group, many felt they were invulnerable to HIV, because HIV was something that happened to other people. The appraisal of their HIV as a threat to their identity meant that these participants needed first to incorporate their diagnosis into their identity or in some way reappraise their sense of self before they could take the next steps of coping effectively with their illness. On average, this group also had elevated levels of depressive mood that remained above the clinical cutoff throughout the year post diagnosis. The work of Baumgartner and colleagues [1,40,57] and Flowers and colleagues[41] demonstrates that the acceptance or incorporation of HIV as a part of one’s identity often comes over time. Although we were able to capture the early HIV appraisals, the present study did not follow participants over the course of years to observe the variability in this integration process. 

In contrast, the participants in the *HIV as Illness* group did not view HIV as a reflection of who they were but as a manageable illness. They acknowledged that HIV was a serious illness that required attention and they intended to take the necessary steps to take control of their care. Their concern for their own health in the wake of the diagnosis was such that they were understandably upset but not overwhelmed by the news and were motivated to do something about it. Overall this group had the lowest levels of depression over the course of the study. This finding is consistent with previous work on illness representations in other diseases which demonstrated that a belief that the disease is controllable was associated with better psychological well-being [19,58]. 

Previous qualitative studies have found HIV illness meanings similar to *HIV as Illness*. For example, Mosack et al[36] discuss a theme of “Restoration of Health” which holds the possibility of improvements in health, despite having HIV. Qualitative studies in samples with illnesses other than HIV report a significant proportion of participants in the “challenge” category [59,60] that may reflect a similar appraisal of the disease as a manageable chronic illness. 

Other studies have described a “wake-up call” or “transformation” appraisal among people living with HIV (e.g.,[26,33,35,36,61]).We did not find strong evidence that this was a dominant appraisal in our sample, who were, on average, within 2 months of diagnosis. It may be that it was too early in the process of adjustment to the diagnosis for participants to have determined that it was a wake-up call. Certainly the stigmatization, concern about dying, and threat to identity groups were struggling with a number of negative consequences of the diagnosis; and it may be that, with time, they would reappraise the news as something that transformed their lives for the better. 

### Limitations

Our sample likely does not reflect the full range of possible HIV illness appraisals. The appraisals that were common in our fairly highly educated, primarily male, gay/bisexual, urban sample may not be reflected in more rural samples with a different risk profile who have less access to health care resources and less social support (e.g., [62]). Furthermore, we grouped individuals based on their dominant appraisal as reflected in their testing narratives. Although this approach resulted in discernible groups that differed in subsequent depression trajectory, it oversimplifies the true nature of illness appraisals which likely change and shift even within short periods of time in response to both intraindividual and external influences. 

In contrast to quantitative measures that explicitly ask about specific components of illness appraisals (e.g., symptoms and labels, consequences, beliefs about timeline) our qualitative approach allowed participants to talk about the most salient aspects of their HIV diagnosis experience, which did not necessarily touch on these pre-determined categories. On one hand, this allows for inclusion of illness appraisals that would not be captured by a quantitative measure. On the other hand, it makes comparison to the large body of literature on illness appraisals more difficult. Future work may capitalize on the strengths of both quantitative and qualitative approaches by including both types of assessments. Our qualitative approach to illness appraisals, while providing rich and detailed data, is time and resource intensive and not practical for translation into a clinical encounter. Future work that maps a qualitative approach onto a briefer measure or assessment would be helpful to providers who wish to incorporate knowledge of illness appraisals into clinical practice. 

We did not look at changes in illness appraisals within individuals over time. It may be that illness appraisals change more rapidly in the initial months after diagnosis. Knowledge of these patterns would help to flesh out our understanding of the process over time and would potentially provide important clues to help engage people in timely and optimal HIV care. Similarly, the data on which the present analyses are based were collected between 2004 and 2008 and it may be that the dominant illness appraisals across individuals have shifted significantly since the data were collected, given the rapidly shifting nature of HIV testing and treatment. Despite the age of the data, we believe the present analyses illustrate the differences (and similarities) in illness appraisals compared to other qualitative work that was done prior to the widespread use of ART. Our group continues to interview people newly diagnosed with HIV and, although the field continues to change rapidly, the data presented here still provide relevant insight into the illness appraisals of people newly diagnosed with HIV. 

Our focus on illness appraisals within a stress and coping theoretical framework did not include other variables that have been shown to predict psychological adjustment to HIV such as social support [63–66], self esteem and self-efficacy [67,68], or optimism [69,70]. These dispositional and contextual factors certainly play a role in the adjustment process and, in fact, are described in relation to appraisals and coping as part of Stress and Coping theory[10]. There is a need for an overarching explanatory model to account for depression in people newly diagnosed with HIV, however, that was beyond the scope of the present work. Instead we attempted to provide a more microscopic view of appraisal that we argue is important to understanding the issues of coping and adjustment to serious illness. We hope that the present work will be incorporated into future studies that attempt to flesh out these more extensive explanatory models that take into account the complexities of the illness adjustment process.

## Conclusions

Posttest counselors and other HIV service providers need to take individual differences in illness appraisals into account in helping newly HIV-positive clients to cope adaptively with their diagnosis, and to engage with and manage their healthcare. People who see HIV as a chronic illness can be reinforced in their proactive approach to seeking care. People whose initial reaction is that HIV is imminently terminal need reassurance and education about new treatment options that are prolonging the lives of people with HIV. Service providers may need to pay particular attention to addressing the needs of those individuals experiencing strong feelings of stigma and those who appraise HIV as a threat to their identity. These people may have difficulties coming to terms with having HIV and may avoid any reminders of their diagnosis. This could negatively influence their ability and motivation to seek necessary care. Finally, providers need to recognize that for some people other issues are perceived as being more pressing than HIV. They will need assistance in dealing with those concerns before they are able to address issues relating to HIV[63]. 
